# Diptera Dwelling Aquatic and Terrestrial Habitats in an Alpine Floodplain (Amola Glacier, Italian Alps)

**DOI:** 10.3390/insects15110904

**Published:** 2024-11-19

**Authors:** Daniele Avesani, Davide Frizzera, Giuseppe Lo Giudice, Daniele Birtele, Valeria Lencioni

**Affiliations:** 1 Natural History Museum of Verona, Lungadige Porta Vittoria 9, I-37129 Verona, Italy; danieleavesani@yahoo.it; 2 Climate and Ecology Unit, Research and Museum Collections Office, MUSE-Museo Delle Scienze of Trento, Corso del Lavoro e Della Scienza 3, I-38122 Trento, Italy; davide.frizzera@uniud.it; 3 La.Na.BIT (Laboratorio Nazionale Tassonomia e Bioindicazione Invertebrati), Raggruppamento Carabinieri Biodiversità, Reparto Biodiversità Verona, Via Tommaso da Vico, 1, I-37123 Verona, Italy; giuseppelogiudice78@gmail.com; 4 Reparto Carabinieri Biodiversità di Roma, via del Canale Della Lingua 74, I-00124 Roma, Italy; d.birtele@gmail.com

**Keywords:** Chironomidae, Muscidae, glacier foreland, Malaise trap, emergence trap, new records, phenology, Alps

## Abstract

Among flying insects, Diptera were the main visitors and colonisers of aquatic and terrestrial habitats in an Alpine glacial floodplain (NE Italy) at 2400 m a.s.l. In all, 4317 dipteran adults were collected using different collection techniques in, on, and out of the water: pond and drift nets, and emergence and Malaise traps, with a different periodicity: biweekly and every three hours for four consecutive days, in early and late summer 2015. Thirty-eight families in all, and 56 species within seven Brachycera families, were identified. Specifically, Chironomidae (36%) within Nematocera and Empidoidea families (23%), and Muscidae (9%) within Brachycera, prevailed. Chironomidae seemed to emerge and fly mainly in late morning–early afternoon, while most Brachycera were more active in late afternoon. Some ecological notes are given for seven Brachycera families, including Muscidae as the predominant family of anthophilous dipterans and the most efficient pollinators in mountain habitats and in the deglaciated areas of the proglacial forelands. Three genera of Muscidae were found as the main representatives of these environments: *Thricops* Rondani, *Spilogona* Schnabl, and *Phaonia* Robineau-Desvoidy). Among these genera, noteworthy was the finding of *Spilogona triangulifera* (Zetterstedt) as being new to the Italian fauna.

## Simple Summary

Among flying insects, Diptera were the main visitors and colonisers of aquatic and terrestrial habitats in an Alpine glacial floodplain (North East Italy) at 2400 m a.s.l. In all, 4317 dipteran adults were collected using different collection techniques in, on, and out of the water: pond and drift nets, and emergence and Malaise traps. Thirty-eight families in all, and 56 species within seven Brachycera families, were identified. Specifically, Chironomidae (36%) within Nematocera and Empidoidea families (23%), and Muscidae (9%) within Brachycera, prevailed. Some phenological and ecological notes were given for Brachycera species. Noteworthy was the finding of *Spilogona triangulifera* (Zetterstedt,) (Muscidae) as being new to the Italian fauna.

## 1. Introduction

Ecosystems at high altitude experience extremely harsh conditions such as low air and water temperature, strong wind, and extended periods of snow cover [1]. The combination of these factors demands, for both aquatic and terrestrial insects, unique physiological adaptation for survival in winter and for development and reproduction in a short time window in summer [2,3]. Specifically, streams flowing in glacier forelands are typically extremely cold, frozen, or covered by snow in winter, turbulent and turbid in summer, and are colonised by few invertebrate taxa, represented mainly by insects and, within them, by the Diptera families Chironomidae, Empididae, Limoniidae, and Simuliidae. Diamesinae (Chironomidae) are the dipterans best adapted to colonise the glacier-fed streams, being able to grow at temperatures ranging from 0 to 4 °C in summer and to survive freezing [2]. Dipterans, with semi-aquatic and terrestrial species (e.g., Muscidae), are also frequent and abundant components of the nival fauna and dwellers of glacier forelands and alpine meadows, also with a role of pollinators [4,5,6]. This is not surprising, considering that the order Diptera, with nearly 160,000 species in approximately 10,000 genera and 158 families [7,8,9], is among the most species-rich insect orders (accounting nearly 15% of the world’s known insect species) [10]. They also include a wide variety of adaptations that reflect their tremendous morphological and ecological diversity and have a worldwide distribution. They play a central role in water purification, matter and energy transfer in riparian ecosystems, carbon cycling in lakes and forests, and decomposition and recycling of organic matter and pollination. They also provide a major prey base for many other invertebrates as well as vertebrates such as amphibians, bats, birds, and fish [11]. In turn, several families contain predators and parasitoids as larvae and adults, including the Dolichopodidae, Empididae, Syrphidae, and Tachinidae [12].

Many taxonomic keys have been published on Diptera, mainly at the adult stage, but few specialists are experienced in attributing them to species [11]. Specifically, knowledge on the dipteran communities living in alpine valleys is still scarce, due also to the need to use multiple methods to collect species with such different autecology.

The aim of this work was to potentially capture all dipteran families, aquatic, semi-aquatic, and terrestrial, that frequent wetlands and meadows above 2000 m altitude in the Alps, in a glacier catchment. We started from the considerations that the emergence time of aquatic species with terrestrial adults ranges from late spring/early summer to early autumn, most of the species are uni- or bivoltine [13], and the plant visited by terrestrial species occurs in the snow-free period [14]. We used different sampling techniques (Malaise and emergence traps, and pond and drift nets) every three hours for three consecutive days, and biweekly, during summer, in a floodplain crossed by a glacier-fed and a spring-fed stream, upstream of their confluence. This work represents one of the most exhaustive surveys on dipteran in a high altitude glacier catchment, adding knowledge on the study area that has been investigated previously from the glaciological, geomorphological, and ecological points of view [5,14,15,16,17,18], and that is a destination for hikers within the Adamello-Brenta Natural Park.

## 2. Materials and Methods

### 2.1. Study Area

**Figure 1:**
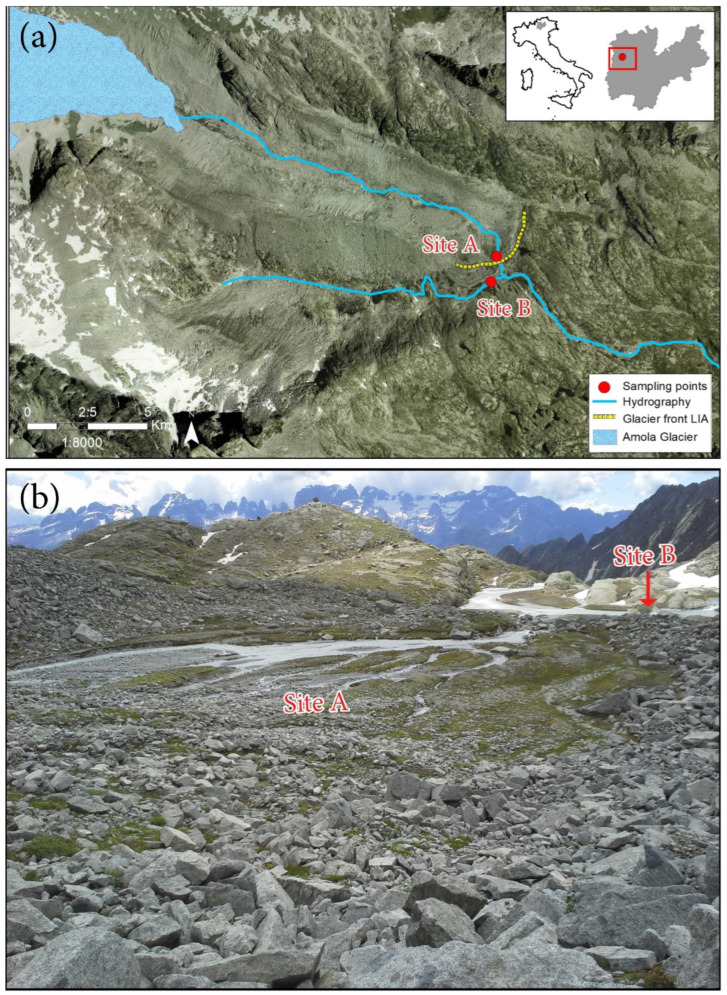
Study area in NE Italy (Trentino Province) and sampling sites (located in the red square): (**a**) map of the glacier foreland of the Vedretta d’Amola glacier according to the regional geocartographic portal (http://www.territorio.provincia.tn.it/portal/server.pt/community/database_geografico_provinciale/1085/database_geografico_provinciale/289311, accessed on 12 November 2024) (courtesy by F. Paoli); (**b**) Amola glacial floodplain.

The study area (N 46°12.776′ E 010°42.404′) is located in the Adamello-Brenta Natural Park (Trentino, NE Italy), in the Adamello-Presanella mountain range (Figure 1). Two sampling sites were selected in the floodplain crossed by the glacial stream-fed lower boundary within the Amola glacier foreland. The glacier foreland, starting at the glacier front at 2567 m a.s.l., is c. 1.3 km long, covers an altitudinal range of c. 130 m, and is characterised by a large moraine system dating back to the Little Ice Age (LIA, c. AD 1850, Figure 1). The glacial sampling site (Site A) was selected at 2421 m a.s.l. on substrata of the LIA moraines, at 1.28 km from the front [18]. This stream is turbulent and turbid in summer, with a substrate dominated by sand, gravel, and cobbles [17]. The non-glacial stream (Site B) is fed by surrounding springs at 2.41 km from the main source, is transparent and meandering, and with silt and sand as the dominant substrate [17]. Site B was selected at 2414 m a.s.l., upstream from the confluence with the glacial stream. The two sites are 87 m distant apart as the crow flies. Air temperature, recorded with an air thermometer during sampling time close to the Malaise trap, ranged from 2.0 to 13.6 °C between 7 July and 26 September, with an average of 7.6 ± 4.9 °C. It was a fairly dry summer, with thunderstorms in late June/early July, late August, and mid-September.

### 2.2. Sampling Schedule

**Figure 2:**
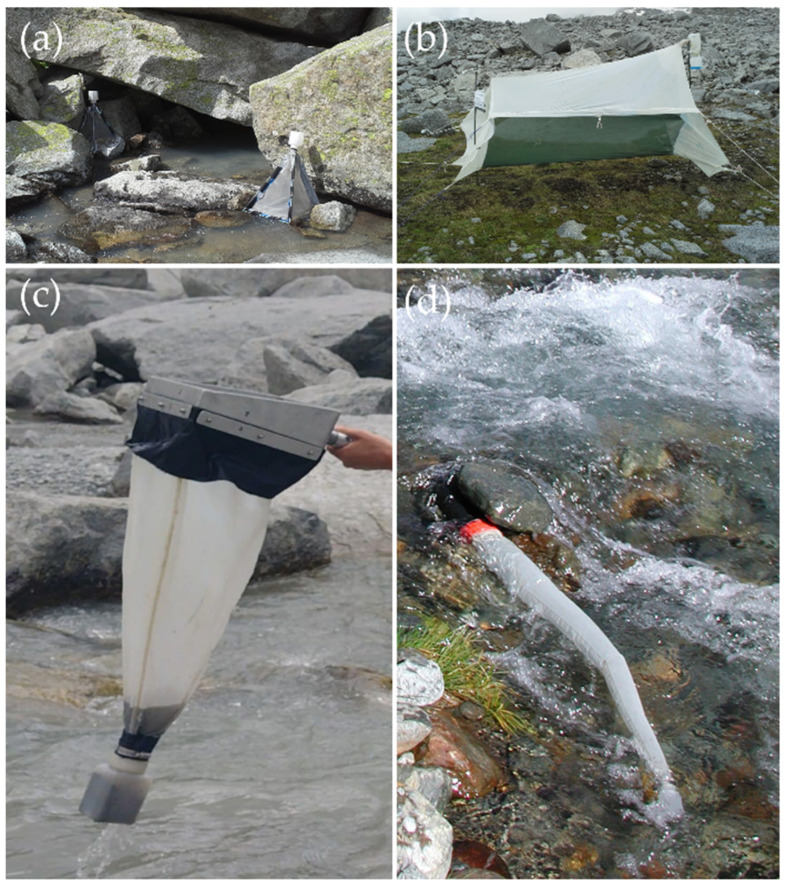
The four sampling methods used: (**a**) emergence trap; (**b**) Malaise trap; (**c**) pond net; (**d**) drift net.

We used different sampling techniques to catch dipteran adults, on and in the water of both sites, using pond and drift nets, and emergence traps, and on the soil close to river banks of the Site A with one Malaise trap (Figure 2). Samples were collected during summer 2015 according to this sampling schedule:

- “biweekly sampling”: approximately every 15 days (at intervals varying from a minimum of 11 to a maximum of 20 days), between 7 July and 26 September, the Malaise and emergence traps were emptied, a kick semi-quantitative sample and one drift sample were collected in each site.
- “time sampling”: occurred twice and lasted 4 days each time: 23–26 July and 10–13 September. The samples were collected every 3 h between 6 AM and 9 PM for a total of 6 samples per day and 18 for each campaign. Time sampling was carried out using the drift net and the two traps.

The choice of dates, periods, and times of collection is based on the authors’ experience of dipteran phenology in mountain environments. Collections were carried out during the snow-free period, every fortnight or so and at different times of day in order to intercept the greatest number of species, active at different times and with a different life cycle duration.

Individuals were preserved in the field in 75% ethanol.

#### 2.2.1. Emergence and Malaise Traps

Emergence traps are cage-like structures used to capture aquatic insects during their transition from late aquatic instar (nymph/subimage/pupa) to terrestrial adults. The trap consists of a floorless, pyramidal, closed tent (rectangular base 42 cm base length, 100 μm mesh size) with a jar containing diluted ethylene glycol (10%) as a killing agent arranged on the highest point. Ethylene glycol, which is the most widely, used preservative because of its slow evaporation and clean preservation of insects collected over long periods [19]. The emergence trap usually is used to estimate the insect population density. Three emergence traps were positioned in each site, anchored to the shore with stakes (Figure 2a).

The Malaise trap is a tend structure used for trapping flying insects, and made of a netting material (Figure 2b). The insects fly into the tent wall and, thanks to their tendency to move upward, are funneled into a collecting jar attached to the highest point. Insects were trapped in the jar partially filled with diluted ethylene glycol (10%) as a preservative. The Malaise trap was placed in the floodplain crossed by the glacial stream, close and perpendicularly to the site A banks, to intercept the highest number of insects moving upstream and between sites A and B.

Both traps were emptied biweekly and every 3 h in the two periods mentioned above.

#### 2.2.2. Pond and Drift Nets

The pond net (frame 30 × 30 cm, 250 μm mesh size) was used to collect benthic fauna by kick sampling, investigating in each site five different microhabitats for a total of 10 min (2 min per microhabitats) exploring a substrate surface of about 0.5 m^2^ in all (Figure 2c). Samples were pooled together, filtered in the field to remove an excess of water and preserved in 75% ethanol. The kick-sampling was performed only biweekly to catch any dead adults fallen in the substrate.

The drift net (diameter 10 cm, 1 m of 100 μm mesh size net) was left against the current in each site upstream of the area where kick samples were collected according to Lencioni [17] (Figure 2d). The drift sampling was performed to catch any alive or dead adults floating in the current from upstream. Drift nets were used (one per site) during the biweekly sampling for 1 h and during the time sampling for 30 min.

### 2.3. Diptera Identification and Data Analysis

All samples were sorted and Diptera adults isolated for identification at family level (using Avesani and Lencioni [12], and Oosterbroek [20]). Adults belonging to seven families of the Brachycera suborder were identified to species level according to: Whittington and Beuk [21], and Beuk [22] (Lonchopteridae); Collin [23], Czerny [24], Gorodkov [25], Papp [26], and Woźnica [27] (Heleomyzidae); Speight and Sarthou [28] (Syrphidae); Merz [29] (Tephritidae); Pont and Meier [30] (Sepsidae); Gregor et al. [31], Hennig [32,33], and Savage [34] (Muscidae; the genitalia of at least one male individual, if present, of each species were extracted and examined).

The collection was deposited at the MUSE-Science Museum of Trento (Entomological collection INV017).

Differences in time and among sampling sites were tested by the Mann–Whitney U test and Analysis of variance (ANOVA Kruskal–Wallis test) using the software STATISTICA 12© StatSoft. Results were considered significant with a *p*-value < 0.05. Ecological notes were given for the 56 identified species.

Missing data: data from Malaise referring to the period 14–26 September were excluded from the analysis because the trap was found overturned on the last collection day (26 September). The emptying of the emergence traps, the Malaise trap, and the drift nets on 24 July at 3 PM, and on 11 September at 6 AM, was not carried out due to bad atmospheric conditions. The same for the emptying of the Malaise trap on 10 September at 6 PM due to destruction of the collecting jar.

## 3. Results

The list and abundance (5135 ind. in all) of the 11 insect orders sorted from all samples collected are reported in Table S1.

### 3.1. Dipteran Fauna Composition

**Figure 3:**
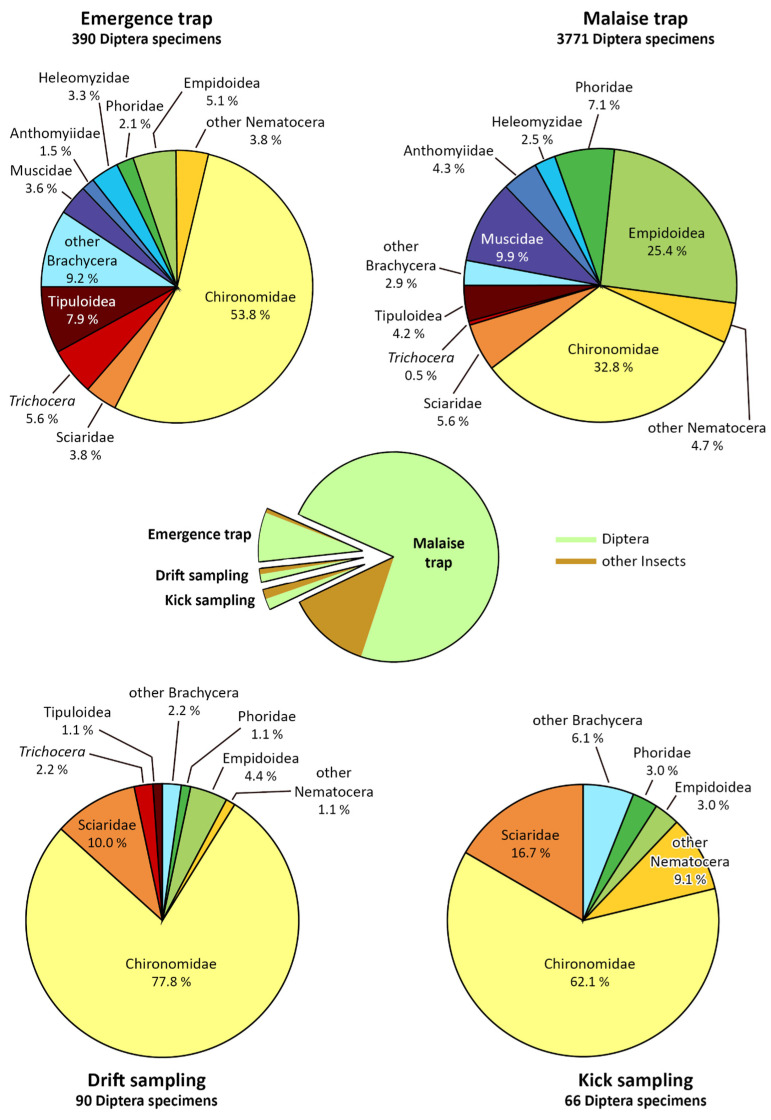
Percent composition of the Diptera adults collected with the emergence traps and the Malaise trap, and with the drift and pond nets. In the middle is the percentage of Diptera and other insect orders collected with the four different techniques. Limoniidae is displayed together with Tipulidae and Pediciidae as the Tipuloidea superfamily; Dolichopodidae is displayed together with Empididae s.l. as the Empidoidea superfamily.

Diptera accounted for 84.1% of all individuals collected, followed by Hymenoptera (10.5%). The percentage of Diptera ranges from 54.2% and 59.5% in drift and kick sampling to 85.1% and 91.3% in the Malaise trap and emergence traps (Figure 3). In all, 4317 dipteran adults were collected using different techniques in, on, and out of the water: kick (111 ind.) and drift (166 ind.) sampling, emergence (427 ind.) and Malaise (4431 ind.) traps (Table S1).

Thirty-eight families were identified. Among these, seven Brachycera families were identified at species level (Lonchopteridae, Syrphidae, Tephritidae, Sepsidae, Heleomyzidae, Muscidae, and Tachinidae) in a list of 56 species including 29 Muscidae and 10 Syrphidae (Table 1 and Table S2). It is to be noted that, in Table 1, the family “Empididae s.l.” (i.e., Empididae *sensu latu*) is understood to be the group of five families that were recognised by Sinclair and Cumming [35] in 2006 as separate taxa: Atelestidae, Hybotidae, Empididae s.str., Brachystomatidae and Microphoridae. Figure 3 summarises the percent composition in detail of the insect that one of the Diptera community collected with the four techniques. In the pie charts, families with the highest number of individuals are displayed, while less represented families are grouped into broader taxonomic categories.

Specifically, Chironomidae (1559 ind., 36%) within Nematocera and Empidoidea families (952 ind., 23%), and Muscidae (386 ind., 9%) within Brachycera, prevailed. In smaller but consistent quantity we found: Phoridae (278 ind., 6.4%), Sciaridae (248 ind., 5.7%), Anthomyiidae (167 ind., 3.9%), Heleomyzidae (106 ind., 2.5%), and Limoniidae (104 ind., 2.4%).

Most individuals and families were captured by the Malaise trap independently of the time schedule. Specifically, all identified families (apart from Asteiidae) were captured with the Malaise. However, for the less abundant families (Limoniidae, Pediciidae, Simuliidae, Dolichopodidae, Lonchopteridae, Sepsidae, Agromyzidae, Drosophilidae, and Ephydridae) the contribution of the emergence traps was not negligible, and for some families was even higher (Sphaeroceridae and Trichoceridae).

**Figure 4:**
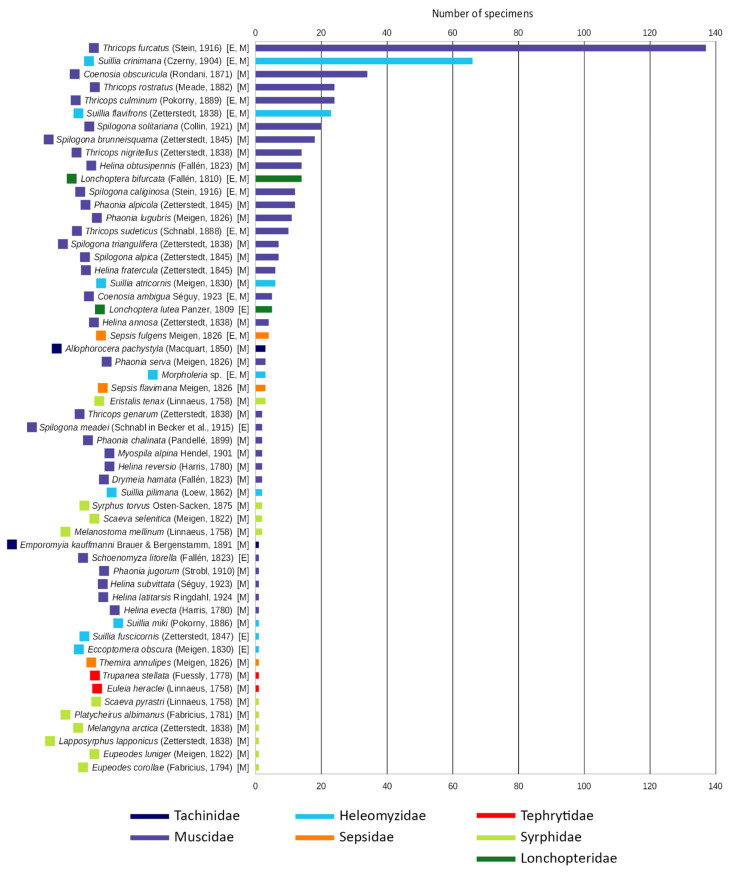
Ranking of the 56 identified species in descending order according to the number of individuals captured for each species. The color of each bar corresponds to the respective families. In square brackets, it is indicated with “E” or “M” if the specimens were captured with emergence traps or Malaise traps, respectively, or with both methods.

All the 530 individuals from the families Lonchopteridae, Syrphidae, Tephritidae, Sepsidae, Heleomyzidae, Muscidae, and Tachinidae, identified at the species level, were collected exclusively using emergence traps and the Malaise trap, as reported in Table S2, Appendix A, and Figure 4.

The family with the highest number of species was by far the Muscidae, with 29 species identified, along with one additional probable species, *Helina* cf. *cinerella*, which could not be identified with certainty and therefore not included in the graphical analysis. This was followed by the Syrphidae with 10 species, the Heleomyzidae with 8 species, including one identified at the genus level (*Morpholeria* sp.), the Sepsidae with 3 species, and the Lonchopteridae, Tephritidae, and Tachinidae all with only 2 species each. The dominance of two species, *Thricops furcatus* (Stein) and *Suillia crinimana* (Czerny), was evident, as they together make up more than one third of all identified individuals, while most species are represented by a small number of individuals: the median value is, in fact, 2.5 individuals.

### 3.2. Seasonal Samplings

#### 3.2.1. Emergence Traps and Malaise Trap

**Figure 5:**
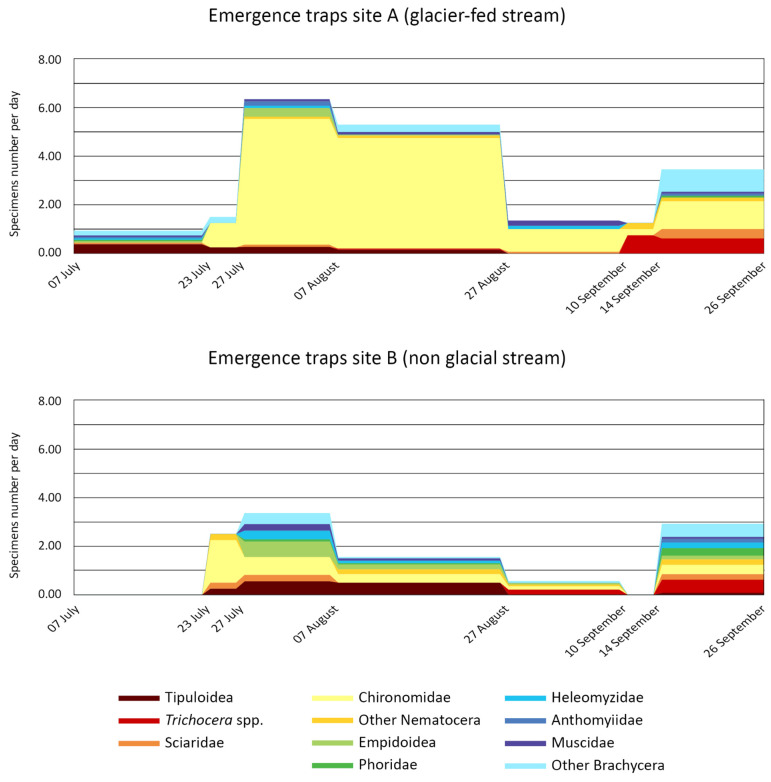
Temporal trend of the individual numbers collected during summer 2015 using the three emergence traps in site A and three in site B. The values are expressed as the average number of individuals/collection day.

**Figure 6:**
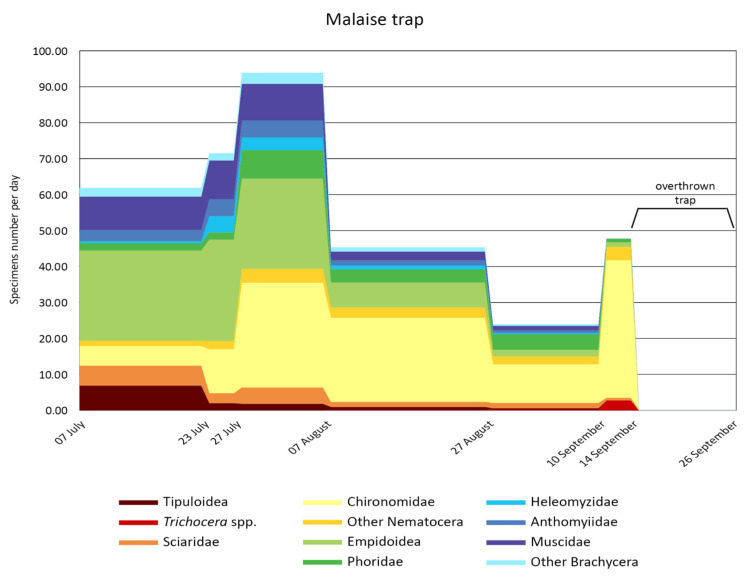
Temporal trend of the individual numbers collected during summer 2015 using the Malaise trap. The values are expressed as the average number of individuals/collection day.

Figure 5 and Figure 6 show the seasonal trend of individuals collected using the emergence and Malaise traps, respectively. In the graphs, we plotted the average number of individuals collected/day from July to September, with each time slot being approximately fortnightly. Additionally, to cover the entire summer period, the time collections performed in the two four-day slots were also included.

The number of individuals collected at the glacial site was almost double that collected at the non-glacial site (Figure 5) in July and August, while the number was comparable in September. Chironomidae significantly (*p* = 0.003) prevailed in the Diptera collection at Site A, where they represented >80% of the benthic and drift communities. Their dominance was not evident in the non-glacial stream where Empidoidea and Tipuloidea were also well represented. Tipuloidea were caught mainly in July and August, while *Trichocera* spp. was almost only in September, in both sites.

Chironomidae again represented the dominant taxon after 27 July. Before this date, Empidoidea and other Brachycera prevailed. As observed with emergence traps, Tipuloidea and Empidoidea were caught significantly (*p* = 0.33) mainly in July and August, while *Trichocera* spp. was at the end of the summer.

#### 3.2.2. Kick Samplings

**Figure 7:**
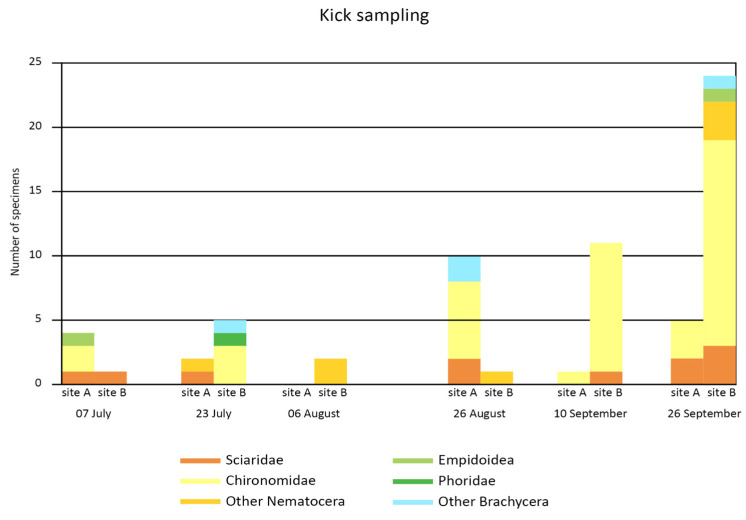
Number of adult Diptera for each family or family groups collected by Kick sampling across the 6 times during the sampling season in the two sites A (glacier-fed stream) and B (non-glacial stream).

Figure 7 shows the relative abundance of adult Diptera collected by kick sampling at the six collection times during summer 2015 in site A and site B. The counted individuals are adult Diptera that fell on the surface of the stream water, presumably at the time of sampling or shortly before, in the sector of the stream upstream from the sampling site.

Due to the low number of individuals, any statistical comparison was hindered among collection times and sites. In any case, it is possible to note that Chironomidae prevailed in both sites over three to four dates, followed by other Nematocera, Sciaridae, and, in the non-glacial stream (site B), also by Psychodidae, Simuliidae, Ceratopogonidae, Phoridae, and other Brachycera.

### 3.3. “Time” Samplings

**Figure 8:**
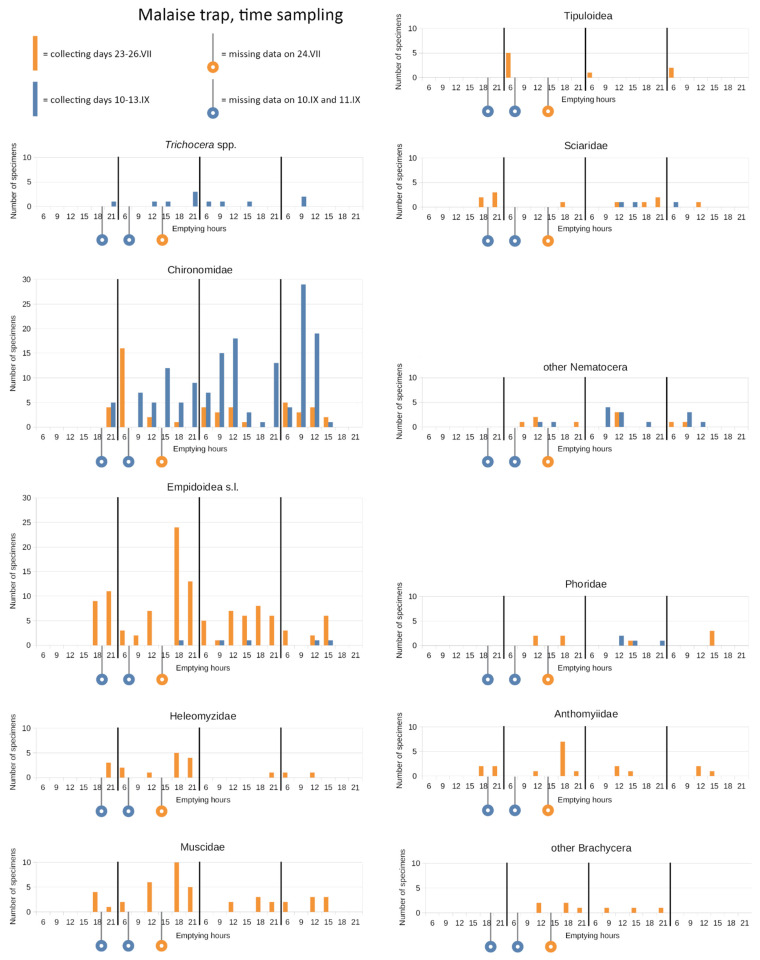
Time sampling of the Malaise trap. The six daily emptying times, namely 6 AM, 9 AM, 12 PM, 3 PM, 6 PM, and 9 PM, are indicated with 6, 9, 12, 15, 18, and 21, respectively. The orange and blue circles indicate the missed emptying on 24 June at 3 PM, and on 11 September at 6 AM, due to bad atmospheric conditions, and the missed emptying on 10 September 2015 at 6 PM, due to destruction of the collecting jar. The results from the two “time sampling“ periods, namely 23–26 July and 10–13 September, are overlaid on the graphs and indicated by two different colors, orange and blue, respectively.

Results referring to emergence traps and drift nets are plotted in Figures S2 and S3. Figure 8 shows results referring to the Malaise trap. Due to the low number of individuals collected with the emergence traps and drift, it is arduous to define a temporal trend for the different taxa. Considering only the dominant ones, Chironomidae seemed to emerge preferentially between midday and 3 PM in Site A, but also at 9 AM in site B. With the drift net, Chironomidae were caught at any hour between 6 AM and 3 PM in site A, while also at 9 PM in site B. Chironomidae were caught by the Malaise trap at any time, with a significantly higher abundance (*p* = 0.04) at between 9 AM and 3 PM in both periods (July and September). In addition, Empidoidea were collected at any time but mainly in July, with significant peaks (*p* = 0.04) in late afternoon (6 PM–9 PM). Even if not significantly, other Brachycera were caught mainly in the late afternoon.

**Figure 9:**
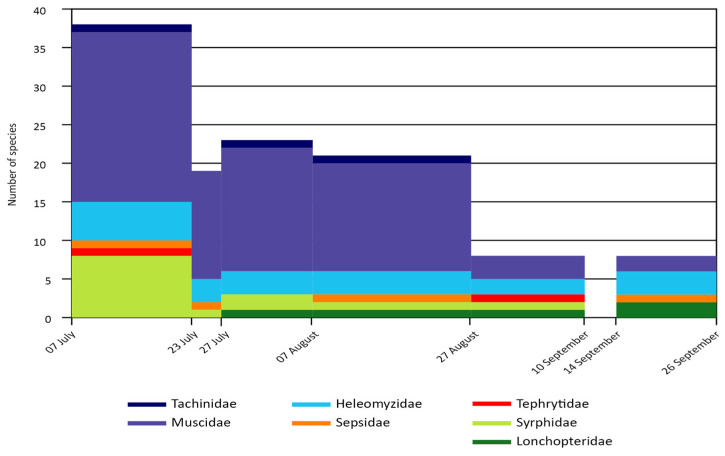
Trend in the number of species during the sampling season related to the seven families of Diptera Brachycera for which individuals were identified at the species level. The results refer to the species captured with both Malaise traps and emergence traps during the ‘biweekly’ and ‘time’ samplings.

Figure 9 shows the seasonal trend in the number of species for these families of Diptera Brachycera. It should be noted that, during the last collection period (14–26 September), the Malaise trap was found overturned, so the reports for this period refer only to the emergence traps.

The period with the highest number of species was the initial one, from 7 to 23 July, during which Muscidae, Syrphidae, and Heleomyzidae showed the greatest biodiversity. The lowest number of species, on the other hand, occurred after 27 August. During the period from 7 July to 27 August, the largest contribution was given by Muscidae.

## 4. Discussion

### 4.1. Diptera Distribution and Phenology

Several studies demonstrated the importance of Diptera in alpine and nival environments as the main insect pollinators, both in Alpine and Arctic environments [4,36,37,38] with a high number of individuals and species. Specifically, they are main colonisers of recently deglaciated areas in the glacier forelands, numerically increasing from earlier to mature successional stages of soil and vegetation. This was highlighted by Losapio et al. [5] and Albrecht et al. [39] for the Amola glacier foreland. Their study, despite the long tradition of dipterological research carried out in the Alps over the past 150 years, continues to yield new knowledge and discoveries, as demonstrated by the results of the “Diptera Stelviana” project in the Stilfserjoch National Park [40,41], reporting 248 new species for the Italian fauna and 10 new species were described for science [40] (pp. 14–15, 360–366). That dipterans turned out to be the dominant insect order in our collections was, therefore, expected, including in more than 4300 individual aquatic, semi-aquatic, and terrestrial species, about half belonging to Nematocera and half to Brachycera. As expected, Chironomidae (Nematocera) and Empidoidea (Brachycera) prevailed, together accounting for 59% of the collected individuals. In fact, both families have aquatic larval stages and are the dominant dipterans in high-altitude streams. Specifically, Chironomidae with the genus *Diamesa* Meigen is the most frequent and abundant taxon colonising glacier-fed and spring-fed streams above 2000 m a.s.l. [17]. Additionally, being aquatic insects with terrestrial adults, they were captured with all sampling methods, traps, and nets in and out of the water. Among Brachycera, Muscidae also include species with aquatic larvae or that are associated with wet habitats in floodplains in any case, such as *Schoenomyza litorella* (Fallén) and six species of the genus *Spilogona* Schnabl, among which are *S. caliginosa* (Stein) and *S. meadei* (Schnabl in Becker et al.) that, in fact, we collected only or also with the emergence traps. Surprisingly the emergence traps, built to catch emerging adults of aquatic/semi aquatic insects from the stream, also caught species or families known as terrestrial. This is the case for the families Sphaeroceridae, Drosophilidae, and Anthomyiidae, and the species *Sepsis fulgens* Meigen (Sepsidae), *Suillia crinimana* (Czerny), *S. flavifrons* (Zetterstedt), *S. fuscicornis* (Zetterstedt), *Eccoptomera obscura* (Meigen), *Morpholeria* sp. (Heleomyzidae), *Coenosia ambigua* Séguy, *Thricops culminum* (Pokorny), *T. furcatus* (Stein), and *T. sudeticus* (Schnabl) (Muscidae). For these individuals, it must, therefore, be assumed that the adult entered the emergence trap during a dry phase of the stream, which allowed entry from the lower part of the trap, or that it emerged from pupae carried by the current during periods of high water in the stream.

Specifically, *Eccoptomera obscura* (Meigen) is a European psychrophilous species with a poorly known biology [42], probably with necrophagous habits [43]. As the congeneric species, it is known to be a troglophilous species and can be found around cave openings in damp and shaded places [44], and sometimes on snow [42]; the species belonging to the genera *Suillia* Robineau-Desvoidy and *Morpholeria* Garrett are known to have, respectively, mycetophagous [45,46] and saprophagous larvae [42].

As expected, the pond net, and so the kick sampling, was also efficient in the collection of Nematocera, predominantly represented by families with aquatic larvae: Sciaridae and Chironomidae, the latter constituting more than 60% of the individuals.

All families apart from one, the Brachycera Asteiidae, were captured with the Malaise that missed; within the 56 identified species, only five taxa were, conversely, taken by the emergence traps. Emergence traps placed close to the riverbanks can, therefore, be valuable tools to supplement collections of adult terrestrial species traditionally collected using Malaise traps [47].

Considering the mode of operation and the size of the collection area, it is expected that the Malaise will have collected several individuals from the emergence traps (even summing the catches of the six traps together), even on single days or time slots of the same day. What was comparable was the seasonality of occurrence for some families. Specifically, Chironomidae were caught during the entire period, while Tipuloidea, Empidoidea, and Muscidae were caught mainly in July and August, when the air temperature was higher (maximum > 10 °C), as expected at high altitude according to Pont [48] (pp. 195–196).

Trichoceridae specimens (all belonging to the genus *Trichocera* Meigen) were collected mainly at the end of the summer, as could be expected. The holarctic genus *Trichocera* Meigen, in fact, like almost all species in the family, is known for its cold adaptation; adults are typically found during the cold season, from early autumn to spring [49,50], and can also be found in cold karst cavities with the presence of ice [51,52]. Due to the low number of individuals collected/hour, it was difficult to define a daily emergence/visiting pattern for the different taxa collected with the traps or drift.

In any case, for a few families we observed a trend with peaks of Empidoidea and other brachyceran families in late afternoon (6 PM–9 PM) as expected. In fact, according to the literature, a significant reduction in the presence and abundance of flower visitor insects (including Diptera) is expected during the central hours of the day in high-altitude grassland environments [4,53]. Chironomidae, the dominant family, were caught by the Malaise trap at any time, with a significantly higher abundance between 9 AM and 3 PM in both periods (July and September), and by the drift between 6 AM and 3 PM. Additionally, the emergence traps suggested they emerge preferentially between midday and 3 PM in Site A but also at 9 AM in site B. So, finally, we can suggest that late morning–early afternoon is the best time slot to catch them. It is interesting to note that an increase in the number of individuals collected with the pond net corresponded to a decrease in the number of emerged specimens. Vice-versa, a decrease in the number of specimens collected with the kick sampling corresponded to an increase in the number of the emergence organisms. This proportionally inverse relation reflects the life cycle of insects.

### 4.2. Ecological Notes of Brachycera Species

The 56 species recorded in the Amola floodplain, belonging to the seven families of brachyceran dipterans that were identified, represent a significantly lower number than what one would expect to find in alpine grasslands at a corresponding altitude ([41], pp. 283–311), [48,54,55]. This can partly be explained by the fact that, although the collection campaign covered almost the entirety of the available flight season, it still focused on a circumscribed and delimited area with specific environmental characteristics. Scarce vegetation was present, dominated by anemophilous species like *Carex curvula* All., representing >80% ground cover [14]. The list includes species with a wide distribution and broad ecological tolerance, which are of little faunistic interest. Among these: some Syrphidae such as *Eristalis tenax* (Linnaeus), *Platycheirus albimanus* (Fabricius), *Eupeodes corollae* (Fabricius), and *E. luniger* (Meigen); some Muscidae such as *Helina evecta* (Harris), *H. reversio* (Harris), and *Schoenomyza litorella* (Fallén); the two species of Lonchopteridae, the very common *Lonchoptera lutea* Panzer, but also *L. bifurcata* (Fallén) [56,57]; the Heleomyzidae, *Suillia fuscicornis* (Zetterstedt), and the three species of the Sepsidae family: *Themira annulipes* (Meigen), *Sepsis flavimana* Meigen, and *S. fulgens* Meigen.

Some species, on the other hand, are typically montane, although more common in forests at lower altitudes, likely passively transported by air currents or actively ascending the slopes during the season. This is the case for: some Syrphidae such as *Melangyna arctica* (Zetterstedt), *Scaeva selenitica* (Meigen), and *Syrphus torvus* Osten-Sacken; some Heleomyzidae such as *Suillia atricornis* (Meigen); several Muscidae: *Drymeia hamata* (Fallén), some species of the genus *Helina* Robineau-Desvoidy: *H. evecta* (Harris), *H. fratercula* (Zetterstedt), and *H. latitarsis* Ringdahl; the genus *Phaonia* Robineau-Desvoidy: *P. jugorum* (Strobl), *P. lugubris* (Meigen), and *P. serva* (Meigen); the genus *Spilogona* Schnabl: *S. brunneisquama* (Zetterstedt), *S. meadei* (Schnabl in Becker), and *S. solitariana* (Collin), in addition to *Thricops genarum* (Zetterstedt) and *T. nigritellus* (Zetterstedt).

Other collected species are, instead, characteristic of high-altitude grassland environments or commonly found above the tree line. In this category, besides *Suillia crinimana* (Czerny) among the Heleomyzidae and the two species of Tachinidae: *Allophorocera pachystyla* (Macquart) and *Emporomyia kauffmanni* (Brauer and Bergenstamm), the Muscidae family dominates: *Coenosia ambigua* Séguy, *C. obscuricula* (Rondani), *Helina annosa* (Zetterstedt), *H. obtusipennis* (Fallén), *H. subvittata* (Séguy), *Myospila alpina* Hendel, *Phaonia alpicola* (Zetterstedt), *P. chalinata* (Pandellé), *Spilogona alpica* (Zetterstedt), *S. caliginosa* (Stein), *S. triangulifera* (Zetterstedt), *Thricops culminum* (Pokorny), *T. furcatus* (Stein), and *T. rostratus* (Meade).

The number of collected species was low for families like **Sirphydae** (10) considering that it is one of the Diptera Brachycera families with the highest number of species—over 6000 species worldwide and over 535 species known in Italy [58]. This is because they are pollinators uncommon in high altitude meadows that remain partly snow-covered for most of the year. In fact, hoverflies are floricultural and the scarcity of flowering herbaceous plants limits their number. Furthermore, some hoverfly species cannot be easily captured by Malaise traps because they fly very low over the vegetation and even more so in extreme environments. The larvae have a heterogeneous diet (predatory zoophagous larvae, phytophagous, aquatic/subacquatic saprophagous and terrestrial saprophagous) and are closely linked to particular habitat types [59]. For these reasons they are excellent environmental bioindicators. The larvae of some species, like *Eupeodes corollae* (Fabricius) that we collected with the Malaise trap, are aphidophagous, and therefore they are useful for parasite control in agricultural environments. Another family, **Tephritidae,** is characterised by the phytophagous lifestyle of their larvae, which develop within various tissues and organs of the host plant. The family includes about 270 European species and 151 species known for Italy [20,60]. Of these, we collected only *Euleia heraclei* (Linnaeus) and *Trupanea stellata* (Fuessly) that develop during their larval stage at the expense of various types of herbaceous plant. *Trupanea stellata* (Fuessly) frequents several genera of the Asteraceae family as host plants, among which are the genera *Artemisia* L., *Leontodon* L., *Senecio* L., and *Hieracium* L. [29]. These genera are present in the flood plain of the study area or 50–100 m higher along the glacier foreland with the following species: *Artemisia genipi* Weber ex Stechm., *Leontodon helveticum* Mérat, and *Senecio carniolicus* Willd [5] (Suppl. Mat.), and several species of the genus *Hieracium* L. (Prosser F., pers. comm.). *Euleia heraclei* (Linnaeus) is known to develop inside plants belonging to the Apiaceae family [29]. Some plants of this family are present in the study area with the following species: *Peucedanum ostruthium* (L.) (also on the moraines), *Laserpitium halleri* Crantz, and *Laserpitium krapfii* Crantz subsp. *gaudinii* (Moretti) Thell. (Prosser F., pers. comm.).

To be mentioned is the record of the family **Heleomyzidae**, including approximately 600 worldwide, of which there are 80 in Italy [61] and 153 species in Europe [62]. In the study we collected 100 individuals belonging to nine species, seven of which are of the genus *Suillia* Robineau-Desvoidy, known to have mycetophagus larvae. The two more abundant species were *S. crinimana* (Czerny) (>60%), a species restricted to alpine and subalpine zones [27,54], and *S. flavifrons* (Zetterstedt), usually present in mountain habitats and considered a boreal-alpine element [63,64]. This species was also recorded in low-altitude locations but characterised by cold microclimates due to thermal inversion [43].

**Lonchopteridae** also account for species common in alpine environments, like the two unique species we found, *Lonchoptera lutea* Panzer and *L. bifurcata* (Fallén), belonging to this family. We expected these results, considering that it is a small family of Lower Cyclorrhapha Diptera with 14 European species, all belonging to the genus *Lonchoptera* Meigen. They are all associated with the presence of water, from coastal areas to high-altitude mountainous areas, with larvae developing on decaying plant material. *Lonchoptera lutea* Panzer is known to be an extremely eurytopic species with aquatic larvae [11,57,65], so we expected to collect them also with emergence traps. Conversely, we did not expect to find three specimens of *Sepsis fulgens* Meigen in the emergence trap samples. In fact, **Sepsidae** are a family with 44 European species, 31 of which are known in Italy [30,66], whose larvae develop in animal or vegetal decaying matter, dung, and excrement among mushrooms in humus-rich soil. The adults can be found on the leaves and flowers of meadows and clearings, or along watercourses but the three species we found (*Themira annulipes* (Meigen), *Sepsis flavimana* Meigen, and *S. fulgens* Meigen) show a wide and broad ecological distribution, and have mainly coprophilous larvae in soil [30,67,68]. The finding of a few **Tachinidae** was expected, being one of the most numerous families of Diptera, with 640 species known from the Italian fauna [69], with species dwelling in flowers of tree plants or on the ground among rocks or mountain scree. Ziegler [41] (283–311) reported 27 species collected by a Malaise trap from an alpine zone at a similar altitude (2315 m a.s.l.) in Stilfser Joch National Park, including the two species we found: *Allophorocera pachystyla* (Macquart) and *Emporomyia kauffmanni* (Brauer and Bergenstamm).

Finally, **Muscidae** is a well-known family (even from a taxonomical point of view) and best associated with a consistent number of species known to the alpine and nival habitats in the Alps [55,70,71], and in the subarctic zone [36]. Muscidae are the predominant family of anthophilous dipterans and the most efficient pollinators in these habitats (mainly with the genera *Thricops* Rondani, *Spilogona* Schnabl, and *Phaonia* Robineau-Desvoidy) and, together with Anthomyiidae, in the deglaciated areas of the proglacial plains [5,6]. Overall, Muscidae, with 593 species known in Europe, occur in many environments, thanks to a highly differentiated larval lifestyle within the family. In Italy, 308 species are present [72], of which 28 were recorded in our campaign. The twenty-ninth species is *Spilogona triangulifera* (Zetterstedt), new to the Italian fauna and the second record for the Alps. In fact, this species, which is exclusive to the arctic habitat, is widespread in northern Europe from Great Britain to Scandinavia, and from European Russia to Siberia (as well as being present in North America), and was found by Pont [55] in the Austrian Alps in the same habitat as in the present study. With this record, the species thus confirms its disjunct arcto-alpine distribution.

In the material collected with an emergence trap, some individuals of *Spilogona caliginosa* (Stein) and *S. meadei* (Schnabl) were found that are associated with mountain streams, as was one specimen of *Schoenomyza litorella* (Fallén), related to the presence of water in various environments [73,74]. Although the aquatic lifestyle of their larvae has not been proven, it is likely that their larval development occurs in water or in the moist sandy soil of the proglacial plain. On the other hand, it is difficult to explain the capture of *Coenosia ambigua* Séguy and the three *Thricops* species with emergence traps, as their larval stages are presumably associated with the soil of grassland environments.

Five species (*Phaonia chalinata* (Pandellé), *Spilogona alpica* (Zetterstedt), *S. triangulifera* (Zetterstedt), *Thricops culminum* (Pokorny), and *T. rostratus* (Meade)) can be considered exclusive to alpine meadows or nival environments above the treeline [55], and the number of collected individuals represents 16.6% of all Muscidae.

Noteworthy are also some species due to their restricted range: first, *Spilogona caliginosa* (Stein), an endemic species of the Alps, of which 12 specimens were collected, as well as *Phaonia chalinata* (Pandellé), endemic to the high-altitude areas of the Alps and Pyrenees, found in only two specimens. Also worth mentioning are the five specimens of *Coenosia ambigua* Séguy, a species limited to the mountainous areas of the Alps, Pyrenees, and Apennines, and the single specimen of *Phaonia jugorum* (Strobl) that is present only in the Alps and other mountainous areas of central Europe [31].

*Thricops furcatus* (Stein) appears to be the predominant Muscidae species in the proglacial plain of Amola Valley, accounting for 36.1% of all identified Muscidae.

Compared to previous studies conducted in the Alps at the same altitudes and in similar environments, the species diversity of Muscidae collected was significantly lower. In fact, Pont [55] reports 55 species of Muscidae from the nival zone and 54 from the alpine zone (Tyrol, Austria), with a total of 70 species present in the two environments at an altitude between 2101 m and 3020 m, and Pont [48] reports 69 species of Muscidae from the alpine zone at 2315 m in the Stelvio National Park (South Tyrol, Italy). This may be due to the presence of the LIA moraine blocks in the area, on which a well-established herbaceous plant community has not yet been established [18].

## 5. Conclusions

Notwithstanding the harsh environmental conditions of the area, mainly due to the constantly low temperature even during summer, a consistent number of individuals and species of Diptera were collected, proving the first list of this area. The environment of the glacier foreland in the Amola valley turns out to be a specific habitat featuring a community of Diptera that differs from those found in alpine meadows at corresponding altitudes in the Alps. In this environment, the Chironomidae family and the Empidoidea group are dominant, with a significant presence of the Muscidae family.

Regarding biodiversity, the results for some of the studied families (Syrphidae, Heleomyzidae, Muscidae, and Tachinidae) show a marked reduction compared to what was previously found in alpine meadows at similar altitudes in the Alps.

Some Nematocera (e.g., Chironomidae) seem to fly in the middle of the day, while many Brachycera do so in late afternoon.

Regarding capture methods, in addition to confirming the importance of the Malaise trap as the main tool for studying Diptera, this study emphasizes the importance of emergence traps as an auxiliary tool that can significantly contribute to the number of species detected in river floodplains.

## Figures and Tables

**Table 1:** The list and abundance of Diptera families caught by traps and nets. In the last column, the number of species identified within seven Brachicera families.

Diptera Groups	Families	Emergence Traps	Malaise Trap	Drift Sampling	Kick Sampling	Identified Species
NEMATOCERA TIPULOMORPHA	Tipulidae	1	62	-	-	-
	Limoniidae	22	81	1	-	-
	Pediciidae	8	17		-	-
	Trichoceridae (*Trichocera* Meigen, 1803)	22	17	2	-	-
OTHER NEMATOCERA	Bibionidae (*Bibio* Geoffroy, 1762)	2	1	-	-	-
	Mycetophilidae	3	61	-	-	-
	Sciaridae	15	213	9	11	-
	Cecidomyiidae	-	2	-	1	-
	Psychodidae	3	64	1	2	-
	Scatopsidae	-	3	-	-	-
	Thaumaleidae	-	36	-	-	-
	Simuliidae	6	10	-	2	-
	Ceratopogonidae	1	2	-	1	-
	Chironomidae	210	1238	70	41	-
LOWER BRACHYCERA	Rhagionidae	-	13		-	-
	Empididae s.l.	13	933	4	2	-
	Dolichopodidae	7	26	-	-	-
	Phoridae	8	267	1	2	-
	Lonchopteridae	7	12	-	-	2
	Syrphidae	-	15	-	-	10
BRACHYCERA ACALYPTRATAE	Psilidae	-	4	-	-	-
	Tephritidae	-	2	-	-	2
	Lauxaniidae	-	1	-	-	-
	Chamaemyiidae	-	2	-	-	-
	Sciomyzidae	-	13	-	-	-
	Sepsidae	3	5	-	-	3
	Agromyzidae	2	7	-	-	-
	Asteiidae	1	-	-	-	-
	Chloropidae	1	7	-	1	-
	Heleomyzidae	13	93	-	-	7
	Sphaeroceridae	9	5	-	1	-
	Drosophilidae	10	10	1	2	-
	Ephydridae	3	6	-	-	-
BRACHYCERA CALYPTRATAE	Scathophagidae	-	3	-	-	-
	Anthomyiidae	6	161	-	-	-
	Muscidae	14	372	-	-	29
	Calliphoridae Rhinophorinae	-	2	-	-	-
	Tachinidae	-	4	-	-	2

## Data Availability

Data are included in the paper (Table 1), in the Supplementary Material (Tables S1 and S2), and in Appendix A.

## Supplementary Materials

The following supporting information can be downloaded at https://www.mdpi.com/article/10.3390/insects15110904/s1: Table S1—number of collecting specimens of different insects orders; Table S2—list of identified species with the total number of collecting individuals; Figure S1—trend of the individual number collected using emergence traps (site A + site B); Figure S2—time sampling of the emergence traps; Figure S3—time sampling with the drift net.

## Appendix A

A list of the 56 species identified in seven Brachycera families. For each identified species, listed in alphabetical order within its own family, the collection data are provided: number of individuals and sex, collection date, and capture method.

**LONCHOPTERIDAE**
RECORDS:

***Lonchoptera bifurcata* (Fallen, 1810)**

26.VII-6.VIII, 1F (emergence trap site B), 6FF (Malaise trap); 6-26.VIII, 5FF (Malaise trap); 26.VIII-10.IX, 1F (Malaise trap); 13-26.IX, 1F (emergence trap site B).

***Lonchoptera lutea* Panzer, 1809**

13-26.IX, 1F (emergence trap site A), 2MM 2FF (emergence trap site B).
**SYRPHIDAE**
RECORDS:

***Eristalis tenax* (Linnaeus, 1758)**

7-23.VII, 1M; 25.VII, (3:00 PM) 1M; 26.VII-6.VIII, 1F (all Malaise trap).

***Eupeodes corollae* (Fabricius, 1794)**

7-23.VII, 1F (Malaise trap).

***Eupeodes luniger* (Meigen, 1822)**

26.VIII-10.IX, 1F (Malaise trap).

***Lapposyrphus lapponicus* (Zetterstedt, 1838)**

7-23.VII, 1F (Malaise trap).

***Melangyna arctica* (Zetterstedt, 1838)**

6-26.VIII, 1F (Malaise trap).

***Melanostoma mellinum* (Linnaeus, 1758)**

7-23.VII, 1M 1F (Malaise trap).

***Platycheirus albimanus* (Fabricius, 1781)**

7-23.VII, 1M (Malaise trap).

***Scaeva pyrastri* (Linnaeus, 1758)**

7-23.VII, 1F (Malaise trap).

***Scaeva selenitica* (Meigen, 1822)**

7-23.VII, 1F; 26.VII-6.VIII, 1F (all Malaise trap).

***Syrphus torvus* Osten-Sacken, 1875**

7-23.VII, 2MM (Malaise trap).
**TEPHRITIDAE**
RECORDS:

***Euleia heraclei* (Linnaeus, 1758)**

7-23.VII, 1F (Malaise trap).

***Trupanea stellata* (Fuessly, 1778)**

26.VIII-10.IX, 1F (Malaise trap).
**SEPSIDAE**
RECORDS:

***Themira annulipes* (Meigen, 1826)**

24.VII, 1F (Malaise trap).

***Sepsis flavimana* Meigen, 1826**

7-23.VII, 1M 2FF (Malaise trap).

***Sepsis fulgens*, Meigen 1826**

6-26.VIII, 1M (Malaise trap), 1F (emergence trap site A); 13-26.IX, 2FF (emergence trap site A).
**HELEOMYZIDAE**
RECORDS:

***Eccoptomera obscura* (Meigen, 1830)**: 13-26.IX, 1M (emergence trap site B).

***Mopholeria* sp**.

7-23.VII, 1F (Malaise trap); 26.VII-6.VIII, 1F (Malaise trap); 13-26.IX, 1F (emergence trap site B).

***Suillia atricornis* (Meigen, 1830)**

7-23.VII, 1F; 26.VII, (6 AM) 1F; 26.VII-6.VIII, 1M, 2FF; 6-26.VIII, 1M (all Malaise trap).

***Suillia crinimana* (Czerny, 1904)**

7-23.VII, 1F (emergence trap site A); 7-23.VII, 1M 4FF (Malaise trap); 23.VII, 2MM 1F (Malaise trap 9 PM); 24.VII, 1F (Malaise trap 12 AM); 24.VII, 2MM 3FF (Malaise trap 6 PM); 24.VII, (9 PM) 4FF (Malaise trap); 25.VII, (9 PM) 1F (Malaise trap); 26.VII-6.VIII, 1F (emergence trap site A), 3FF (emergence trap site B), 7MM 14FF (Malaise trap); 6-26.VIII, 18 FF (Malaise trap), 2FF (emergence trap site B); 26.VIII-10.IX, 1F (emergence trap site A).

***Suillia flavifrons* (Zetterstedt, 1838)**

7-23.VII, 1F (Malaise trap); 24.VII, (6 AM) 1M 1F (Malaise trap); 26.VII, (12 AM) 1F (Malaise trap); 26.VII-6.VIII, 4MM 9FF (Malaise trap); 26.VIII-10.IX, 1M 4F (Malaise trap); 26.VIII-10.IX, 1F (emergence trap site A).

***Suillia fuscicornis* (Zetterstedt, 1847)**

13-26.IX, 1M (emergence trap site B).

***Suillia mikii* (Pokorny, 1886)**

7-23.VII, 1M (Malaise trap).

***Suillia pilimana* (Loew, 1862)**

6-26.VIII, 2FF (Malaise trap).

***Suillia* sp.**

6-26.VIII, 2FF (Malaise trap); 26.VII-6.VIII, 1F (emergence trap site B).
**MUSCIDAE**
RECORDS:

***Coenosia ambigua* Séguy, 1923**

7.VII-23.VII, 2MM 1F (Malaise trap); 26.VII-6.VIII, 1M (emergence trap site A); 6-26.VIII, 1F (Malaise trap).

***Coenosia obscuricula* (Rondani, 1871)**

7.VII-23.VII, 8MM 18FF; 23.VII, (6 PM) 1F; 24.VII, (12:00 AM) 1M; 25.VII, (12 AM) 1M; 1F; 26.VII-6.VIII, 1M 3FF; 6-26.VIII, 1F (all Malaise trap).

***Drymeia hamata* (Fallén, 1823)**

24.VII, (12 AM) 1M; 25.VII, 1M (all Malaise trap).

***Helina annosa* (Zetterstedt, 1838)**

26.VII-6.VIII, 1M 3FF (Malaise trap).

***Helina evecta* (Harris, 1780)**

6-26.VIII, 1F (Malaise trap).

***Helina fratercula* (Zetterstedt, 1845)**

7-23.VII, 1M; 24.VII, (6 AM) 1F; 26.VII-6.VIII, 1M 1F; 6-26.VIII, 1F; 26.VIII-10.IX, 1M (all Malaise trap).

***Helina latitarsis* Ringdahl, 1924**

7-23.VII, 1F (Malaise trap).

***Helina obtusipennis* (Fallén, 1823)**

7-23.VII, 4MM 4FF; 23.VII, (6 PM) 1F; 24.VII, (12 AM) 1F; 26.VII-6.VIII, 2FF; 6-26.VIII, 2MM (all Malaise trap).

***Helina reversio* (Harris, 1780)**

7-23.VII, 1M 1F (Malaise trap).

***Helina subvittata* (Séguy, 1923)**

7-23.VII, 1M (Malaise trap).

***Helina* cf. *cinerella***

26.VII-6.VIII, 1F; 6-26.VIII, 1F (all Malaise trap).

***Helina* sp.**

6-26.VIII, 1F (Malaise trap).

***Myospila alpina* Hendel, 1901**

26.VII-6.VIII, 1M; 6-26.VIII, 1M (all Malaise trap).

***Phaonia alpicola* (Zetterstedt, 1845)**

7-23.VII, 4MM 7FF; 24.VII, (6:00 PM) 1F (all Malaise trap).

***Phaonia chalinata* (Pandellé, 1899)**

7.VII-23.VII 2MM (Malaise trap).

***Phaonia jugorum* (Strobl, 1910)**

26.VII-6.VIII, 1M (Malaise trap).

***Phaonia lugubris* (Meigen, 1826)**

7-23.VII, 4MM 4FF; 26.VII-6.VIII, 2MM; 6-26.VIII, 1F (all Malaise trap).

***Phaonia serva* (Meigen, 1826)**

7-23.VII, 2FF; 26.VII, (3 PM) 1M (all Malaise trap).

***Schoenomyza litorella* (Fallén, 1823)**

13-26.IX, 1F (emergence trap site A).

***Spilogona alpica* (Zetterstedt, 1845)**

7-23.VII, 2MM 3FF; 6-26.VIII, 1M 1F (all Malaise trap).

***Spilogona brunneisquama* (Zetterstedt, 1845)**

7-23.VII, 2MM 12FF; 26.VII-6.VIII, 4FF (all Malaise trap).

***Spilogona caliginosa* (Stein, 1916)**

7-23.VII, 1M 4FF (Malaise trap); 24.VII, (6:00 PM) 1M 1F (Malaise trap); 26.VII, 1F (Malaise trap); 26.VII-6.VIII, 1F (emergence trap site B), 2FF (Malaise trap); 6-26.VIII, 1M (Malaise trap).

***Spilogona meadei* (Schnabl in Becker et al., 1915)**

6-26.VIII, 1F; 26.VIII-10.IX, 1F (all emergence trap site A).

***Spilogona solitariana* (Collin, 1921)**

7-23.VII, 3MM 2FF; 24.VII, (6 PM) 1F; 26.VII, (6:00 AM) 1M, (12 AM) 1M, (3 PM) 1F; 26.VII-6.VIII, 6MM 5FF (all Malaise trap).

***Spilogona triangulifera* (Zetterstedt, 1838)**

7-23.VII, 2MM 5FF (Malaise trap).

***Thricops culminum* (Pokorny, 1889)**

7-23.VII, 8MM 5FF (Malaise trap); 24.VII, (6:00 PM) 2FF (Malaise trap); 25.VII, (6 PM) 1F (Malaise trap); 26.VII-6.VIII, 4FF 1M (Malaise trap), 1F (emergence trap site B); 6-26.VIII, 2FF (Malaise trap).

***Thricops furcatus* (Stein, 1916)**

7-23.VII, 1F (emergence trap site A), 13MM 11FF (Malaise trap); 23.VII, (6 PM) 2FF (Malaise trap); 24.VII, (6 AM) 1F, (12 AM) 1M 1F, (6 PM) 1F, (9 PM) 2MM 3FF (Malaise trap); 25.VII, (9 PM) 2FF (Malaise trap); 26.VII, (12 AM) 1F (Malaise trap); 26.VII-6.VIII, 19MM 28FF (Malaise trap); 6-26.VIII, 1F (emergence trap site A), 1F (emergence trap site B), 6MM 21FF (Malaise trap); 26.VIII-10.IX, 2FF (emergence trap site A), 1M 16FF (Malaise trap); 13-26.IX, 1F (emergence trap site B), 2FF (Malaise trap).

***Thricops genarum* (Zetterstedt, 1838)**

7-23.VII, 1M; 23.VII, (9 PM) 1M (all Malaise trap).

***Thricops nigritellus* (Zetterstedt, 1838)**

7-23.VII, 3MM; 24.VII, (12 AM) 1M, (6 PM) 2FF; 25.VII, (6 PM) 1M; 26.VII-6.VIII, 2MM 2FF; 6-26.VIII, 1M 2FF (all Malaise trap).

***Thricops rostratus* (Meade, 1882)**

7-23.VII, 4FF; 24.VII, (6 PM) 1F; 26.VII-6.VIII, 16FF; 6-26.VIII, 3FF (all Malaise trap).

***Thricops sudeticus* (Schnabl, 1888)**

7-23.VII, 1F (Malaise trap); 25.VII, (12 AM) 1F (Malaise trap); 26.VII, (6 AM) 1F, (12 AM) 1F (Malaise trap); 26.VII-6.VIII, 1F (emergence trap site B), 1MM 4FF (Malaise trap).

***Thricops* sp.**

26.VII-6.VIII, 1F (Malaise trap).

***Thricops* sp.**

6-26.VIII, 1F (Malaise trap).

**Muscidae gen. sp.**

7-23.VII, 1F (Malaise trap); 6-26.VIII, 1F (emergence trap site B).
**TACHINIDAE**
RECORDS:

***Allophorocera pachystyla* (Macquart, 1850)**

7-23.VII, 1M 1F; 26.VII-6.VIII, 1F (all Malaise trap).

***Emporomyia kauffmanni* Brauer & Bergenstamm, 1891**

6-26.VIII, 1M (Malaise trap).
