# Supplementary material for: Diptera Dwelling Aquatic and Terrestrial Habitats in an Alpine Floodplain (Amola Glacier, Italian Alps)
Source: Insects. 2024 Nov 19;15(11):904. doi: 10.3390/insects15110904 (PMC11595050; doi:10.3390/insects15110904)
Supplement: Supplementary file 1 [file insects-15-00904-s001.zip › insects-3240466-supplementary.pdf]

Table S1. List of orders of collected insects with number of collecting specimens and with the number of specimens for each different capture method.

	Emergence traps	Malaise trap	Drift samplings	Kick samplings	Total number of specimens	%
Ephemeroptera	2	-	-	-	**2**	**0,0**
Orthoptera	-	1	-	-	**1**	**0,0**
Plecoptera	18	30	-	1	**49**	**1,0**
Psocoptera	-	2	6	-	**8**	**0,2**
Thysanoptera	-	5	10	11	**26**	**0,5**
Hemiptera	-	27	29	14	**70**	**1,4**
Neuroptera	-	20	-	-	**20**	**0,4**
Coleoptera	16	68	-	-	**84**	**1,6**
Lepidoptera	-	20	-	-	**20**	**0,4**
Diptera	390	3771	90	66	**4317**	**84,1**
Hymenoptera	1	487	31	19	**538**	**10,5**
**Total**	**427**	**4431**	**166**	**111**	**5135**	

Table S2. List of identified species with the total number of collecting individuals and with the number of individuals for each different capture method.

Family	Species	Total no. of individuals	Capture method (no. individuals)	
			Emergenc e traps	Malaise trap
LONCHOPTERIDAE	*Lonchoptera bifurcata* (Fallén, 1810)	**14**	2	12
	Lonchoptera lutea Panzer, 1809	**5**	5	-
SYRPHIDAE	*Eristalis tenax* (Linnaeus, 1758)	**3**	-	3
	Eupeodes corollae (Fabricius, 1794)	**1**	-	1
	Eupeodes luniger (Meigen, 1822)	**1**	-	1
	Lapposyrphus lapponicus (Zetterstedt, 1838)	**1**	-	1
	Melangyna arctica (Zetterstedt, 1838)	**1**	-	1
	Melanostoma mellinum (Linnaeus, 1758)	**2**	-	2
	Platycheirus albimanus (Fabricius, 1781)	**1**	-	1
	Scaeva pyrastri (Linnaeus, 1758)	**1**	-	1
	Scaeva selenitica (Meigen, 1822)	**2**	-	2
	Syrphus torvus Osten-Sacken, 1875	**2**	-	2
TEPHRITIDAE	*Euleia heraclei* (Linnaeus, 1758)	**1**	-	1

	Trupanea stellata (Fuessly, 1778)	**1**	-	1
SEPSIDAE	*Themira annulipes* (Meigen, 1826)	**1**	-	1
	Sepsis flavimana Meigen, 1826	**3**	-	3
	Sepsis fulgens Meigen, 1826	**4**	3	1
HELEOMYZIDAE	*Eccoptomera obscura* (Meigen, 1830)	**1**	1	-
	Morpholeria sp.	**3**	1	2
	Suillia atricornis (Meigen, 1830)	**6**	-	6
	Suillia crinimana (Czerny, 1904)	**66**	8	58
	Suillia flavifrons (Zetterstedt, 1838)	**23**	1	22
	Suillia fuscicornis (Zetterstedt, 1847)	**1**	1	-
	Suillia miki (Pokorny, 1886)	**1**	-	1
	Suillia pilimana (Loew, 1862)	**2**	-	2
	Suillia sp.	**3**	1	2
MUSCIDAE	*Coenosia ambigua* Séguy, 1923	**5**	1	4
	Coenosia obscuricula (Rondani, 1871)	**34**	-	34
	Drymeia hamata (Fallén, 1823)	**2**	-	2
	Helina annosa (Zetterstedt, 1838)	**4**	-	4
	Helina evecta (Harris, 1780)	**1**	-	1
	Helina fratercula (Zetterstedt, 1845)	**6**	-	6
	Helina latitarsis Ringdahl, 1924	**1**	-	1
	Helina obtusipennis (Fallén, 1823)	**14**	-	14
	Helina reversio (Harris, 1780)	**2**	-	2
	Helina subvittata (Séguy, 1923)	**1**	-	1
	Helina cf. *cinerella*	**2**	-	2
	Helina sp.	**1**	-	1
	Myospila alpina Hendel, 1901	**2**	-	2
	Phaonia alpicola (Zetterstedt, 1845)	**12**	-	12
	Phaonia chalinata (Pandellé, 1899)	**2**	-	2
	Phaonia jugorum (Strobl, 1910)	**1**	-	1
	Phaonia lugubris (Meigen, 1826)	**11**	-	11
	Phaonia serva (Meigen, 1826)	**3**	-	3
	Schoenomyza litorella (Fallén, 1823)	**1**	1	-
	Spilogona alpica (Zetterstedt, 1845)	**7**	-	7

	Spilogona brunneisquama (Zetterstedt, 1845)	**18**	-	18
	Spilogona caliginosa (Stein, 1916)	**12**	1	11
	Spilogona meadei (Schnabl in Becker et al., 1915)	**2**	2	-
	Spilogona solitariana (Collin, 1921)	**20**	-	20
	Spilogona triangulifera (Zetterstedt, 1838)	**7**	-	7
	Thricops culminum (Pokorny, 1889)	**24**	1	23
	Thricops furcatus (Stein, 1916)	**137**	6	131
	Thricops genarum (Zetterstedt, 1838)	**2**	-	2
	Thricops nigritellus (Zetterstedt, 1838)	**14**	-	14
	Thricops rostratus (Meade, 1882)	**24**	-	24
	Thricops sudeticus (Schnabl, 1888)	**10**	1	9
	Thricops sp.	**2**	-	2
	Muscidae indet.	**2**	1	1
TACHINIDAE	*Allophorocera pachystyla* (Macquart, 1850)	**3**	-	3
	Emporomyia kauffmanni Brauer & Bergenstamm, 1891	**1**	-	1

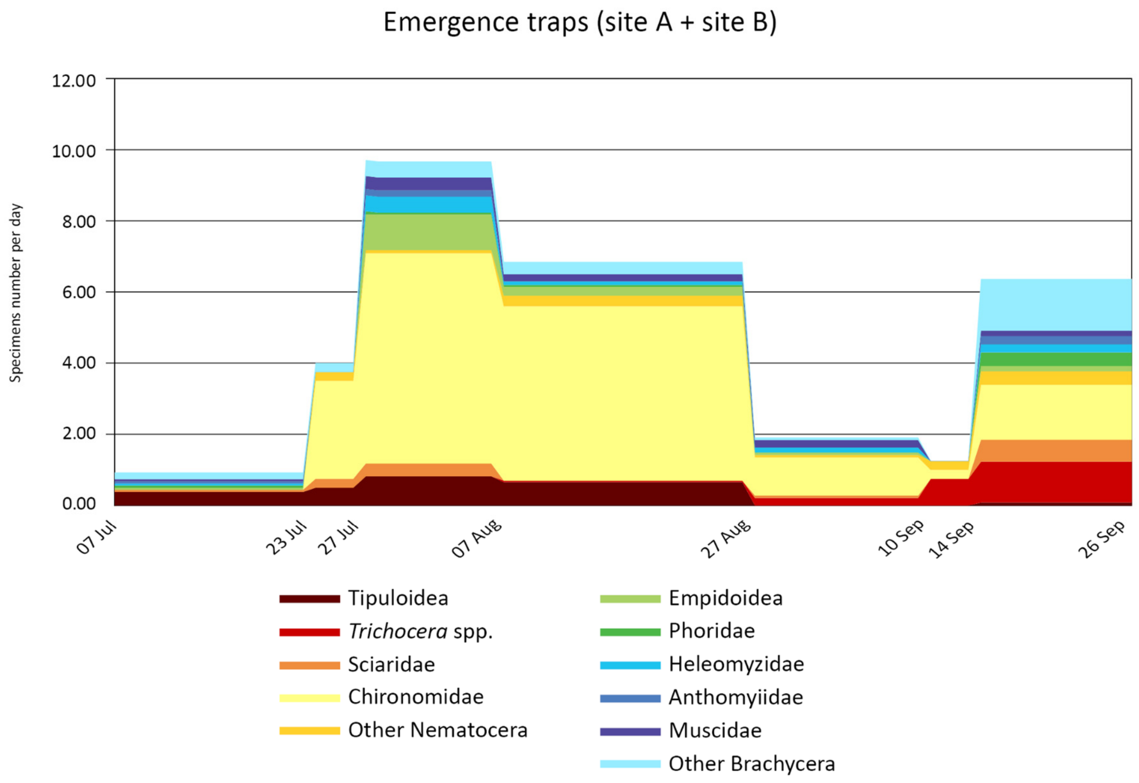

Figure S1. Trend of the individual number collected during summer 2015 using the six emergence traps (site A + site B). The values are expressed as the average number of individuals per collection day.

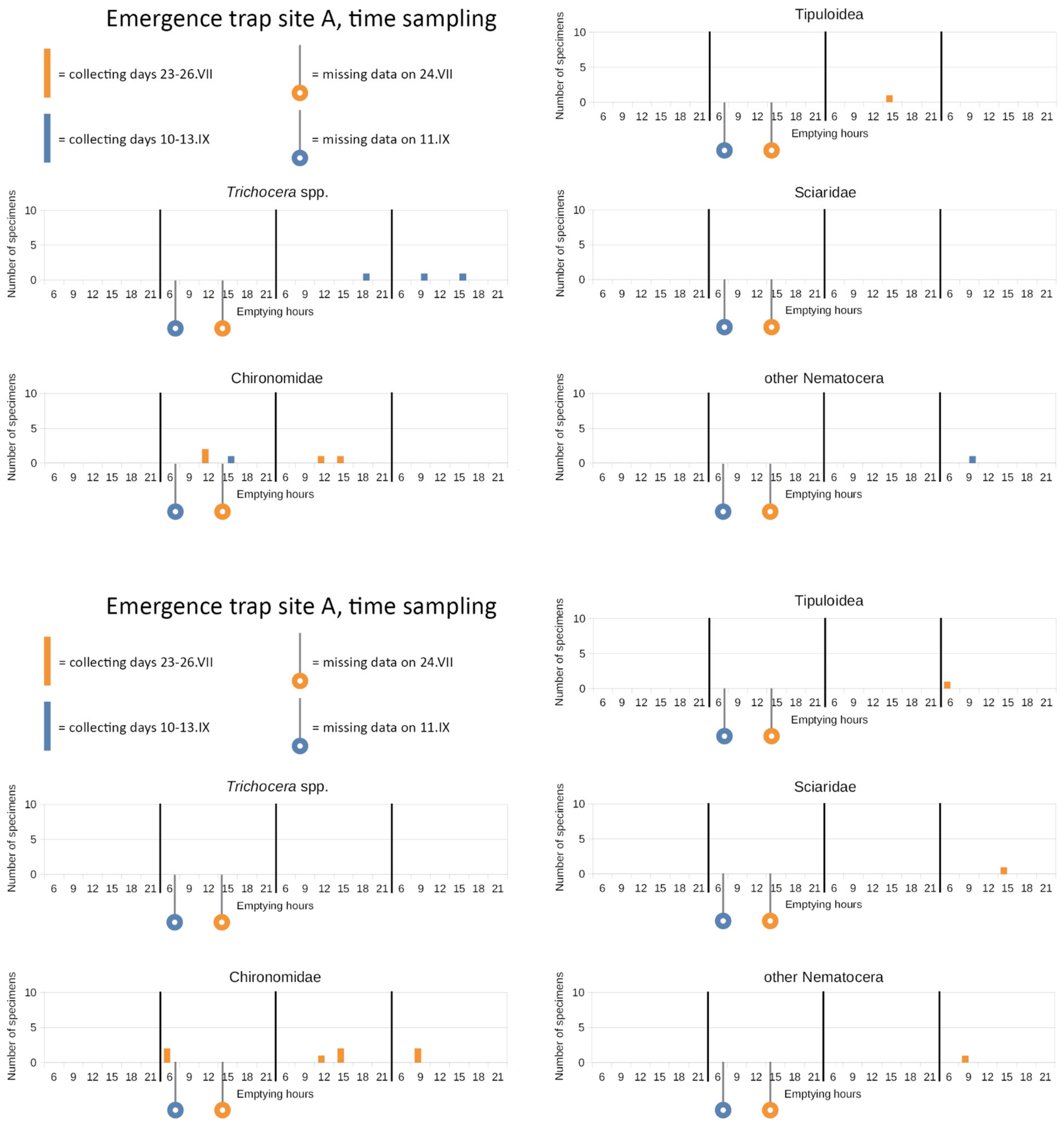

Figure S2. Time samplings with emergence traps at the two sampling sites. The six daily emptyings times, namely 6 AM, 9 AM, 12 PM, 3 PM, 6 PM, and 9 PM, are indicated with 6, 9, 12, 15, 18 and 21 respectively. The orange and blue circles indicated the missed emptyings on June 24, at 3 PM, and on September 11, at 6 PM, due to bad atmospheric conditions. The results from the two "time sampling " collection periods, namely July 23-26 and September 10-13, are overlaid on the graphs and indicated by two different colors, orange and blue, respectively.

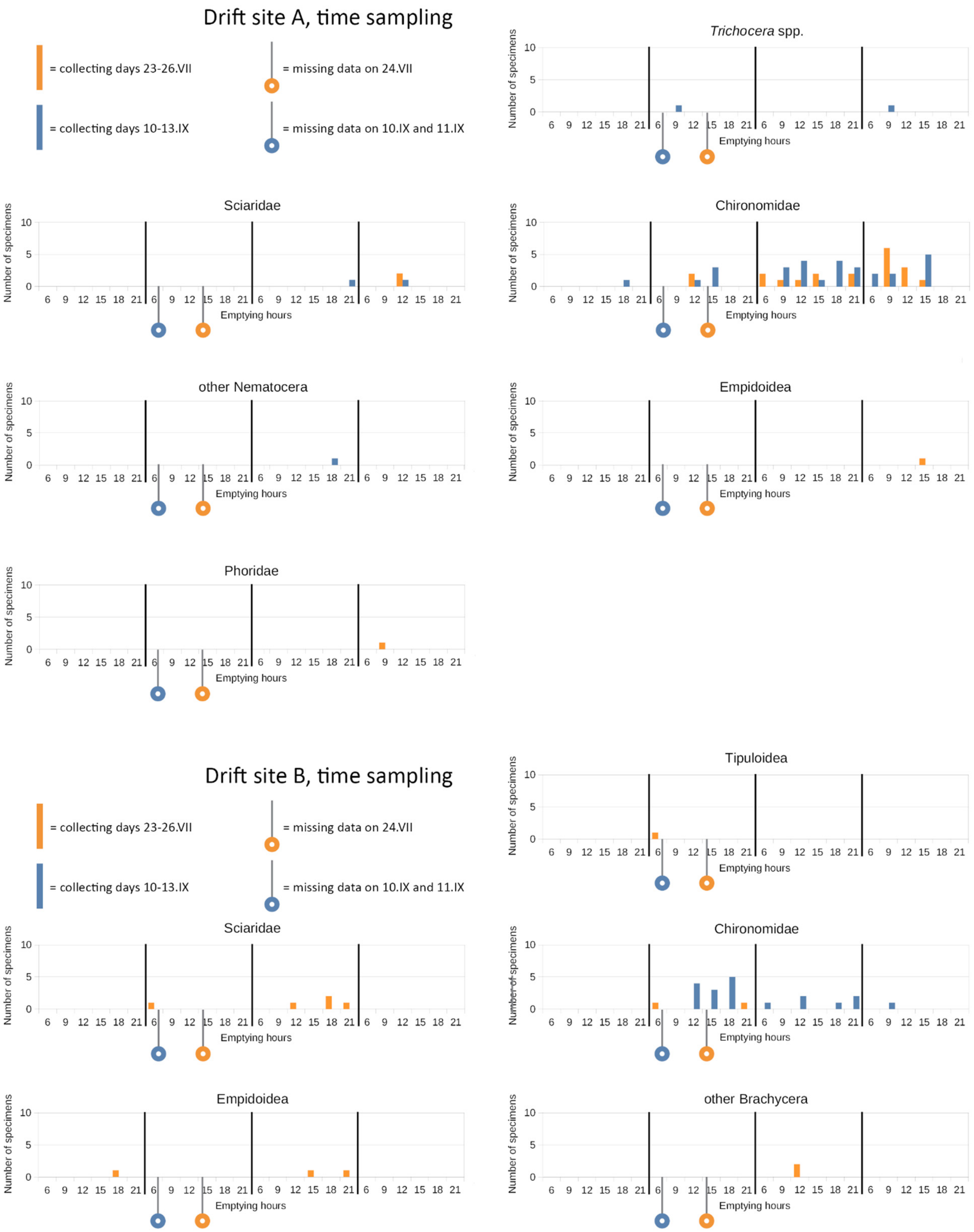

Figure S3. Time samplings with drift samplings at the two sampling sites. The six daily emptyings times, namely 6 AM, 9 AM, 12 PM, 3 PM, 6 PM, and 9 PM, are indicated with 6, 9, 12, 15, 18 and 21 respectively. The orange and blue circles indicated the missed emptyings on June 24, at 3 PM, and on September 11, at 6 PM, due to bad atmospheric

conditions. The results from the two "time sampling " collection periods, namely July 23-26 and September 10-13, are overlaid on the graphs and indicated by two different colors, orange and blue, respectively.
